# Transplantation of A2 type astrocytes promotes neural repair and remyelination after spinal cord injury

**DOI:** 10.1186/s12964-022-01036-6

**Published:** 2023-02-16

**Authors:** Jie Chang, Zhanyang Qian, Binyu Wang, Jiang Cao, Sheng Zhang, Fan Jiang, Renyi Kong, Xiao Yu, Xiaojian Cao, Lei Yang, Hongtao Chen

**Affiliations:** 1 Department of Orthopedic Surgery, Nanjing Drum Tower Hospital, The Affiliated Hospital of Nanjing University Medical School, 321 Zhongshan Road, Nanjing, 210008 Jiangsu China; 2 Department of Orthopedics, The First Affiliated Hospital of Nanjing Medical University, 300 Guangzhou Road, Nanjing, 210029 Jiangsu China; 3 Spine Center, Zhongda Hospital of Southeast University, Nanjing, Jiangsu China; 4 Department of Orthopedics, Taizhou People’s Hospital, Nanjing Medical University, No. 366 Taihu Road, Taizhou, 225300 Jiangsu China; 5 School of Biomedical Engineering and Informatics, Nanjing Medical University, Nanjing, Jiangsu China

**Keywords:** A2 astrocyte, Neural repair, Remyelination, Spinal cord injury

## Abstract

**Background:**

Limited progress in terms of an effective treatment for spinal cord injury (SCI) emphasizes the urgent need for novel therapies. As a vital central nervous system component, the resident astrocytes play crucial roles in regulating recovery after SCI. In this study, recovery after SCI was compared following the transplantation of either A1 or A2 astrocytes. A1 astrocytes are harmful as they upregulate the neurotoxic classical complement cascade genes. Conversely, A2 astrocytes are characterized as neuroprotective as they upregulate the production of many neurotrophic factors.

**Methods:**

We used different supernatant obtained from microglia stimulated with lipopolysaccharide or interleukin-4 to generate A1 and A2 astrocytes. We detected the influence of astrocytes on neurons by co-culturing A1 and A2 astrocytes with neurons. We transplanted astrocytes into the lesion site of the spinal cord and assessed lesion progression, neural restoration, glia formation and locomotor recovery.

**Results:**

Astrocytes were polarized into A1 and A2 phenotypes following culture in the supernatant obtained from microglia stimulated with lipopolysaccharide or interleukin-4, respectively. Furthermore, co-culturing A2 astrocytes with neurons significantly suppressed glutamate-induced neuronal apoptosis and promoted the degree of neuron arborization. Transplantation of these A2 astrocytes into the lesion site of the spinal cord of mice significantly improved motor function recovery, preserved spared supraspinal pathways, decreased glia scar deposition, and increased neurofilament formation at the site of injury compared to the transplantation of A1 astrocytes. Additionally, enhanced A2 astrocytes with potentially beneficial A2-like genes were also detected in the A2 group. Moreover, luxol fast blue staining and electron microscopy indicated increased preservation of myelin with organized structure after transplantation of A2 astrocytes than of A1 astrocytes.

**Conclusions:**

A2 astrocyte transplantation could be a promising potential therapy for SCI.

**Graphical Abstract:**

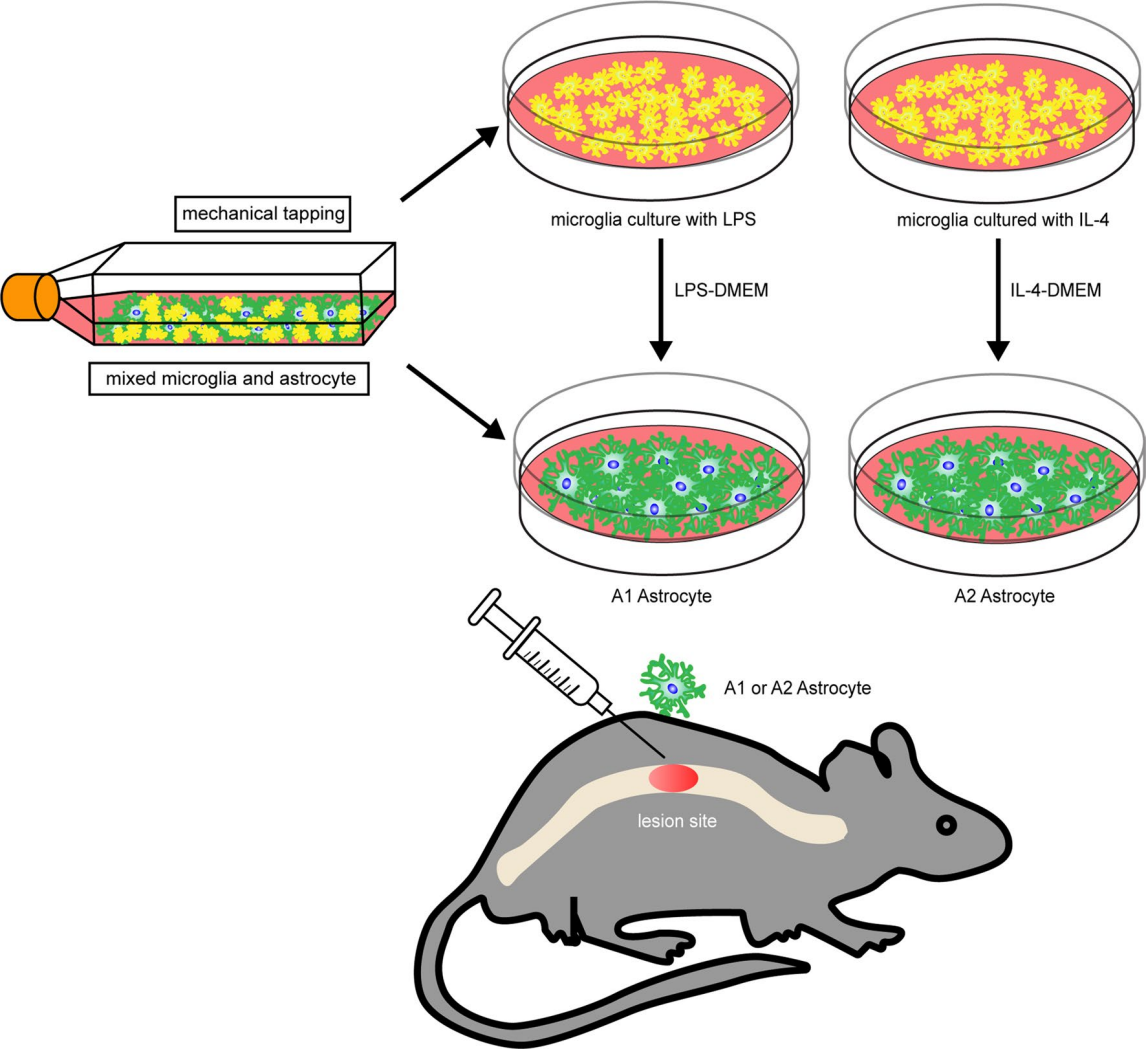

**Video abstract**

**Supplementary Information:**

The online version contains supplementary material available at 10.1186/s12964-022-01036-6.

## Background

Spinal cord injury (SCI) is a devastating disease that can impair neurons and axons, resulting in sensory and motor deficits and an increased risk of paralysis [1, 2]. The incidence of traumatic SCI is documented to be 10.5 cases per 100,000 persons worldwide, placing a huge burden on society globally [3–5]. Despite its severe impact on personal and social life, there is limited effective treatment for SCI [6–8]. The objective of managing SCI is to decompress the spinal cord, achieve early stability, and regain homeostasis while resolving secondary complications [9–11].

SCI lesions comprise three diverse lesion-associated tissue elements: (1) central non-neural lesion core; (2) astrocyte scar boundary; and (3) spared but reactive neural tissue. During the formation of the second element, astrocytes form a border restricting neuroinflammation to protect the adjacent viable neural tissue [12]. During the development of the final element of the SCI lesion, the glial scar develops into a barrier that prevents axonal regeneration [13, 14]. However, astrocytes also express various molecules that promote axon growth following SCI, suggesting that astrocytes aid in axon regeneration [15].

Recently, two types of reactive astrocytes induced by neuroinflammation and ischemia, defined as A1 and A2 astrocytes, have been found to play different roles in the central nervous system (CNS) recovery and repair [16]. A1 type astrocytes are implicated in the destruction of neurons and oligodendrocytes. They are unable to differentiate when directly cultured with lipopolysaccharide (LPS) but are activated when cultured in the medium obtained from LPS-stimulated microglial cultures. A1 astrocytes also overproduce many classical neurotoxic genes resulting in synapse destruction [17]. In contrast, A2 astrocytes release numerous neuroprotective cytokines and regulate brain homeostasis [18, 19]. Therefore, we used Dulbecco's Modified Eagle Medium (DMEM) from microglia activated by LPS or IL-4 to differentiate astrocytes into A1 or A2 astrocytes, respectively. Transplantation of microglia [20], Schwann cells [21], neural stem/progenitor cells [22], and mesenchymal stem cells [23] have been used to improve neurodegenerative conditions. However, as crucial participants in tissue repair, the transplantation of astrocytes has never been investigated as a potential treatment for SCI. In the study, we validated the potential therapeutic effect of A2 astrocyte transplantation on improved motor recovery and myelination after SCI.

## Methods

### Primary cell isolation and treatment

The Ethics Committee of Nanjing Medical University approved the study for scientific research (IACUC-2108026). C57BL6/J and neonatal mice were acquired from the Experimental Animal Center of Nanjing Medical University. Neonatal mice were then sacrificed and immersed in 75% alcohol for 5 min. The dura mater from the head was removed, and the cerebral cortex from the brain tissue was stripped and placed in cold phosphate-buffered saline (+ 5% fetal bovine serum; Gibco, NY, USA). Under an anatomical microscope, the pia meninges and blood vessels on the cerebral cortex surface were removed using micro-anatomical tweezers and washed with cold phosphate-buffered saline (+ 5% fetal bovine serum) 3 times. The enzyme papain was added (KeyGen, Nanjing, China) for tissue digestion at 37 °C for 30 min, and the digestion was terminated with 10% Dulbecco's Modified Eagle Medium/Nutrient Mixture F-12 (DMEM/F12; KeyGen, Nanjing, China). For neuronal extraction, neurobasal medium containing B27 and glutamine (Gibco) was used to resuspend the cell mix, which was cultured for 4 h. Then, the medium was replaced to remove suspended cells and, neurons were allowed to adhere to poly-l-lysine-treated wells. For microglia and astrocyte isolation, a 100 μm cell sieve (WHB, Shanghai, China) was used to filter the cell suspension; the strained cells were then cultured with 10% DMEM/F12 at 37 °C under 5% CO_2_. After 14 days of culture, microglial cells were shaken down, following which the upper layer contained microglia, whereas the lower layer contained astrocytes. Microglia were stimulated with LPS at 40 ng/mL (Sigma, MO, USA) or IL-4 (interleukin -4) at 40 ng/mL (PeproTech, New Jersey, USA) for 3 days. The cellular supernatants termed LPS-activated DMEM or IL-4-activated DMEM from microglia after stimulation were used to culture astrocytes for 3 days. To explore the direct influence of differentiated astrocytes on neurons, we established a co-culture model by using Transwell chamber inserts with 0.4-μm filters (Corning, New York, NY, USA). Neurons were incubated in the lower chamber with 500 μL neurobasal medium for 24 h and polarized astrocytes were cultured in the upper chamber filled with 500 μL 10% DMEM. A 100 μM glutamate (GLU) solution was used to induce excitotoxicity.

### Cell counting kit (CCK)-8 assay

The suitably treated astrocytes were cultured in a 96-well plate (Corning, New York, USA) at 1 × 10^4^ cells/well and incubated for 24 h. Cells were incubated in the CCK-8 test reagent (KeyGen, Nanjing, China) for 4 h, and the absorbance of each well was measured at 450 nm.

### Transwell and scratch wound assay

Astrocytes, after stimulation, were cultured in Transwell chamber inserts with 8-μm filters (Corning, New York, USA). The upper chamber was filled with 2 × 10^5^ cells/mL in serum-free medium, and the lower chamber was filled with 500 μL 10% DMEM. After 24 h culture, unmigrated astrocytes on the upper chamber of the membrane were eliminated and migrated astrocytes on the lower surface were fixed with 4% paraformaldehyde for 15 min and stained with 0.1% crystal violet for 20 min. The scratch wound assay was performed by culturing the treated astrocytes in 2-well inserts (Ibidi, Martin Reid, Germany) in 24-well plates (Corning, New York, NY, USA). The cells migrating into the gap were counted at 24 h.

### Flow cytometry assay

The neuronal apoptotic rate was examined using the Annexin V- fluorescein isothiocyanate/PI apoptosis kit (Multi Sciences, Hangzhou, China) according to the manufacturer’s instructions. The pretreated neurons were incubated with Annexin V and propidium iodide at room temperature in the dark for 5 min. F4/80 (a marker of microglia; 1:500; BD Biosciences, New Jersey, USA) and ACSA-2 (a marker of astrocyte; 1:50; Miltenyi Biotec, Teterow, Germany) were used to detect the purity of Primary cell. Acquired data were analyzed using FlowJo software.

### Western blot

Protein expression of the A1 astrocyte marker C3 (complement 3) and A2 astrocyte marker S100A10 was determined by western blot. Total protein was extracted from astrocytes using the whole cell lysis kit (Keygen, Nanjing, China), and protein concentration was determined using the BCA assay (Thermo Fisher Scientific, New York, USA). Equal amounts of proteins per sample were separated by sodium dodecyl sulphate–polyacrylamide gel electrophoresis and then transferred to polyvinylidene fluoride membranes (Millipore, Massachusetts, USA). The blots were then incubated with primary antibodies against C3 (1:50; Abcam, Cambridge, UK), S100A10 or glyceraldehyde 3-phosphate dehydrogenase (GAPDH; 1:1000; Proteintech, Wuhan, China).

### Immunofluorescent staining

After blocking the unbound sites with bovine serum albumin, the microglial, astrocyte or spinal cord sections were incubated with the primary antibodies against glial fibrillary acidic protein (GFAP, 1:500; CST, MA, USA), S100A10 (1:100; roteintech, Wuhan, China), ionized calcium-binding adapter molecule 1 (IBA-1, 1:500), NF 200 (1:200), C3 (1:50), microtubule-associated protein 2 (MAP2; 1:10,000) and neuronal nuclear protein (NeuN; 1:100; all from Abcam, Cambridge, UK) overnight at 4 °C. Then, the samples were incubated with secondary antibodies Alexa 488 and Alexa 594 (1:200; Invitrogen, New York, USA) for 1 h at room temperature, and the nuclei were stained with 4′,6-diamidino-2-phenylindole fluoromount-G (Southern Biotech, Birmingham, USA). Cells or areas positive for the presence of IBA-1, GFAP, NF-200, C3, and S100A10 were quantified by ImageJ software ImageJ (NIH, Bethesda, MD, USA).

### Neuron morphology analysis

A Sholl analysis of neurons was performed to evaluate the branching complexity according to previous research [24]. Briefly, we drew a series of concentric circles with the neuronal cell body as the center (excluding the cell body) and obtained the number of intersections/crossings of neurons that changed with the distance from the cell body, thus reflecting the complexity of neurons.

### RNA isolation and RT-PCR

Total RNA was extracted from astrocytes using Trizol in accordance with the manufacturer’s instructions (Yifeixue Biotechnology, Nanjing, China), and reverse transcription was conducted using Prime Script TM Master Mix (Takara, Kusatsu, Japan). Real-time PCR was performed using SYBR Green Mix (Vazyme Biotech, Nanjing, China) and the primers were obtained from Genscript (Nanjing, China). The following primers were used: *Fbln5*, forward, 5′- CTTCAGATGCAAGCAACAA-3′ and reverse, 5′- AGGCAGTGTCAGAGGCCTTA-3′; *Serping1*, forward, 5′- ACAGCCCCCTCTGAATTCTT-3′ and reverse, 5′- GGATGCTCTCCAAGTTGCTC-3′; *Srgn*, forward, 5′- GCAAGGTTATCCTGCTCGGA-3′ and reverse, 5′- TGGGAGGGCCGATGTTATTG-3′; *Clcf1*, forward, 5′-CTTCAATCCTCCTCGACTGG-3′ and reverse, 5′-TACGTCGGAGTTCAGCTGTG-3′; *Tgm1*, forward, 5′-CTGTTGGTCCCGTCCCAAA-3′ and reverse, 5′-GGACCTTCCATTGTGCCTGG-3′; *Emp1*, forward, 5′- GAGACACTGGCCAGAAAAGC-3′ and reverse, 5′- TAAAAGGCAAGGGAATGCAC-3′; *GAPDH*, forward, 5′- AAGAGGGATGCTGCCCTTAC-3′ and reverse, 5′- TACGGCCAAATCCGTTCACA-3′. *GAPDH* expression was employed as an internal control. The 2^ΔΔCt^ method was employed to analyze the relative expression.

### Establishment of the SCI model and cell transplantation

Female C57BL/6 J mice aged 6–8 weeks and weighing 20–25 g were anesthetized with ketamine and xylazine via intraperitoneal injection. First, the skin was disinfected and incised, and laminectomy was performed at the T10 level. The T10 spinal cord of the mice underwent moderate contusion injury with an impactor (RWD, Shenzhen, China) (10 g × 20 mm). The mice were randomly assigned into four groups in accordance with the treatment methods: (1) the control group, in which the mice underwent laminectomy without contusion; (2) the injury group, in which the mice underwent laminectomy with contusion and were injected with hydrogel (PEPROTECH, New Jersey, USA) alone; (3) A1 group, in which the mice were administrated 5 μL of hydrogel containing approximately 1 × 10^5^ A1 astrocytes transplanted immediately after SCI; (4) A2 group, in which the mice were treated with 5 μL of hydrogel containing 1 × 10^5^ A2 astrocytes transplanted instantly following SCI.

### Analysis of the locomotion function

A footprint analysis was conducted as previously described [25]. The forelimbs were dipped in red and hindlimbs in blue. We also used the swimming score to evaluate locomotor performance as previously reported [26]. The score is assigned according to the following indices: (0–5 points) hindlimb movements; (0–2 points) hindlimb/forelimb harmonization; (0–1 points) tail position; (0–1 points) paw position; and (0–1 points) sagittal and coronal balance. The basso mouse scale (BMS) score and hindlimb reflex scoring were detected at 1, 3, 7, 14, 21, and 28 dpi, according to a previous study [20]. Two independent investigators observed each mouse for 10 min and recorded the score.

### Electromyography

The electromyography (EMG) signals of gastrocnemius muscle were detected by installing stimulating electrodes in the motor cortex according to a previous study at 6 weeks post SCI [27]. The recording electrode was placed on the gastrocnemius muscle. Signals were obtained by AC amplifier (A-M Systems, WA) and analyzed by LabChart 8.0.

### Visualization of the damaged area

Longitudinal hematoxylin and eosin (H & E) staining was conducted to visualize the damaged area 7 days after SCI and the histologic score [28] was measured 28 days after SCI. The anesthetized mice were placed in a prone position for mice magnetic resonance imaging (MRI) examination (Bruker BioSpec 7 T/20 USR; Bruker AXS GmbH, Karlsruhe, Germany). The sequence procedure was conducted as previously described [29]. The axial plane images were obtained by ParaVision (version 6.0.1, Bruker BioSpec).

### Evaluation of remyelination by LFB staining and electron microscopy

Areas 1 mm above and below the lesion area of the spinal cord were isolated and embedded in paraffin dividing into 3 μm thick sections. We isolated areas 1 mm above and below the lesion area of the spinal cord and embedded them in paraffin dividing them into 3 μm thick sections. The sections were stained with luxol fast blue (LFB) and sealed with neutral resin. The images were observed under microscopy (Olympus, Tokyo, Japan). Six of 30 serial paraffin sections were analyzed in each analysis. Electron microscopy was carried out on spinal cord sections of SCI mice at 28 dpi. Briefly, spinal cords were perfused with 3% paraformaldehyde and 1% glutaraldehyde and samples were observed at 70 nm by transmission electron microscope (FEI Tecnai G2 Spirit Bio TWIN, NY, USA). For G-ratio analysis, at least 100 fibers of each mouse were measured [30].

### Statistical analysis

All data are presented as mean ± SD. We compared two groups by two-tailed unpaired Student’s *t*-test and analyzed the differences among three or more groups via one-way ANOVA followed by Tukey’s post hoc test. SPSS Statistics for Windows, version 20.0 software (IBM Corp., Armonk, NY, USA) was employed to conduct statistical analyses. A *p* < 0.05 was considered statistically significant.

## Results

### Proliferation and migration of astrocytes after polarization

**Fig. 1:**
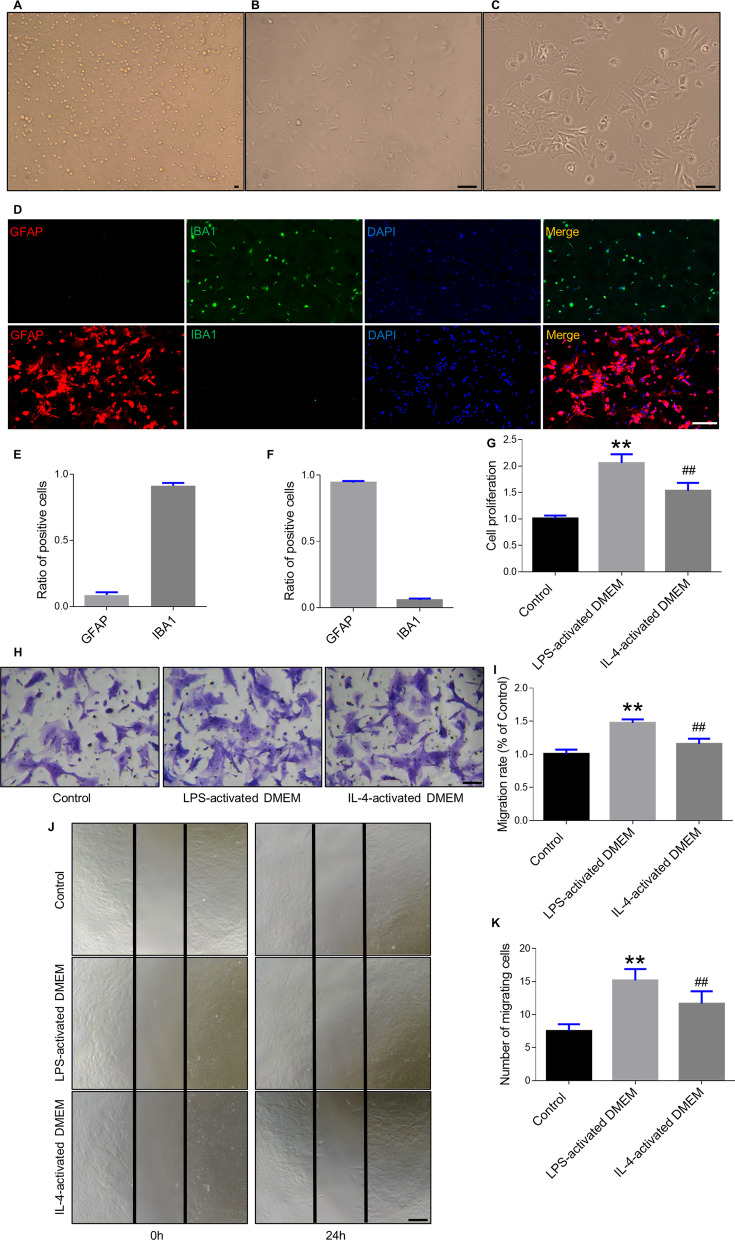
Isolation, proliferation, and migration of astrocytes. **A** Mixed glial cells from the brain cortex. Scale bar = 10 μm. **B** Microglia collection after mechanical shaking. Scale bar = 10 μm. **C** Reserved astrocytes on the bottom. **D** Identification of microglia and astrocyte by IBA-1 and GFAP immunofluorescent staining. Scale bar = 200 μm. Percentage of IBA-1 and GFAP positive cells in microglia (**E**) and astrocyte (**F**). **G** Proliferation assay on astrocytes after culture with LPS-activated DMEM or IL-4-activated DMEM for 3 days. **H** Representative images of vertical migration by the Transwell experiment after treatment. Scale bar = 10 μm. **I** Quantification of (**H**): migration rate. **J** Representative images of horizontal migration by the scratch wound assay. Scale bar = 10 μm. **K** Quantification of (**J**): number of migrating cells. Error bars show means ± SD (n = 3 in each group). **p < 0.01, ^##^p < 0.01 compared to control group

Mixed cells from the cerebral cortex were obtained from newborn mice. After culture for 14 days in DMEM, microglia and astrocyte cells showed proliferation along with stratification (Fig. 1A). Mechanical tapping was used to separate astrocytes from microglia after a 2-week culture. Microglia were rod-shaped with fewer protuberances and astrocytes were star-shaped with many long branches from the cell body (Fig. 1B, C). Immunofluorescence staining analysis of ionized calcium-binding adapter molecule 1 (IBA-1), a pan-microglial marker, and glial fibrillary acidic protein (GFAP) further confirmed the characteristics of microglia and astrocytes (Fig. 1D–F). Flow cytometry assay was also used to determine the purity of the cells (Additional file 1: Fig. S1). In the present study, we found that LPS could not directly polarize astrocytes (Additional file 2: Fig. S2). Therefore, the culture supernatant from LPS- or interleukin (IL)-4-activated microglia was used to induce astrocyte differentiation into A1 or A2 astrocytes, respectively. The astrocyte number increased significantly in the presence of LPS-activated DMEM (DMEM from microglia stimulated by LPS) and IL-4-activated DMEM (DMEM from microglia treated with IL-4) than in cytokine-free DMEM (Fig. 1G). A transwell chamber assay was conducted to quantify the migrated astrocytes. We found that compared to DMEM alone (control), the culture supernatant from treated microglia revealed a significantly increased migration effect (Fig. 1H, I). Wound healing experiments were done to quantify the number of astrocytes migrating into the cell-free gaps. As expected, the number of migrating cells markedly increased in the LPS-activated and IL-4-activated DMEM groups than in the control group (Fig. 1J, K).

### Identification of astrocyte type after in vitro induction

**Fig. 2:**
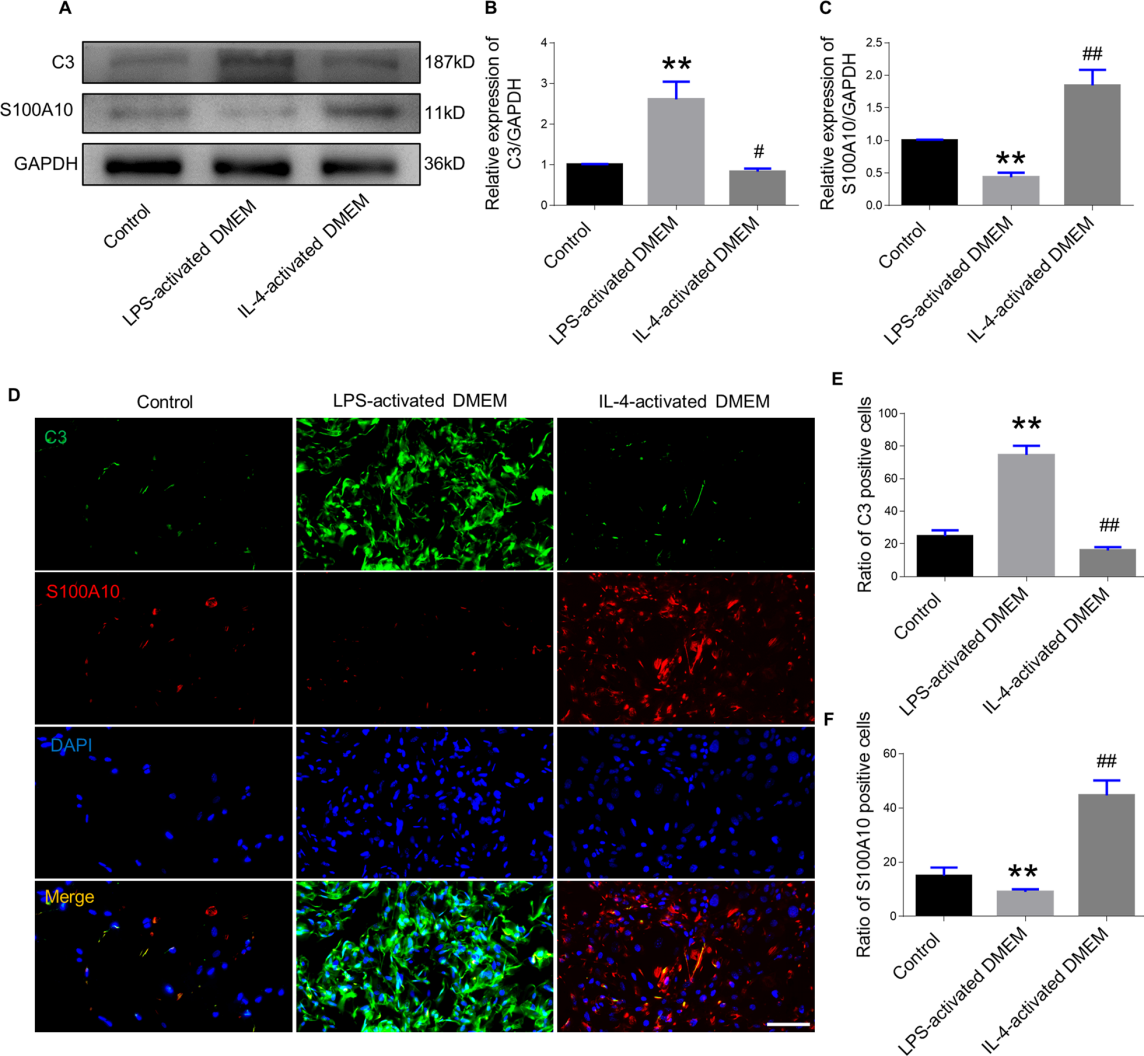
Analysis of A1 and A2 markers of astrocytes after LPS-activated DMEM or IL-4-activated DMEM culture. **A** Western blot analysis of C3 (as an A1 astrocyte marker) and S100A10 (as an A2 astrocyte marker) after astrocytes were induced to differentiate. **B**, **C** Quantification of (**A**). **D** Immunofluorescence staining for C3 and S100A10 in control, LPS-activated DMEM, and IL-4-activated DMEM group on day 3. Scale bar = 100 μm. **E** Quantification of C3-positive cells. **F** Quantification of S100A10-positive cells. Error bars show mean ± SD (n = 3 in each group). **p < 0.01, ^##^p < 0.01 compared to control group

Astrocytes were incubated with LPS-activated DMEM or IL-4 activated DMEM from microglia for 3 days and allowed to differentiate; this was verified by their expression of C3 (an A1 marker) or the S100 protein family member S100A10 (an A2 marker). Significantly higher C3 protein was detected in astrocytes cultured with LPS-activated DMEM than in those cultured with IL-4-activated DMEM and control groups. In contrast, astrocytes stimulated with IL-4-activated DMEM displayed significantly increased S100A10 expression than those stimulated with LPS-activated DMEM and control groups (Fig. 2A–C). Immunofluorescence staining for C3 and S100A10 was conducted to further confirm the effect of culture supernatant from the stimulated microglia on the polarization of the astrocytes. Approximately, 75% of astrocytes stained positive for C3 in the LPS-DMEM group, whereas there were significantly more S100A10 positive cells in the IL-4-DMEM group (Fig. 2D–F). The culture supernatant from microglia can induce differentiation of astrocytes into A1 or A2 types. We next determined whether LPS-activated DMEM promoted neurotoxic gene expression in A1 astrocytes or IL-4-activated DMEM enhanced the release of neuroprotective agents from A2 astrocytes. Reverse transcription-polymerase chain reaction (RT-PCR) was employed to detect three A1-specific neurotoxic gene markers (*Fbln5, Serping1,* and *Srgn*) and three A2-specific gene markers (*Clcf1, Tgm1,* and *Emp1*). Results demonstrated that in the LPS-activated DMEM group, significant upregulation of *Fbln5*, *Serping1,* and *Srgn* expression but a marked reduction of *Clcf1* and *Emp1* expression than in the control group was observed (Additional file 3: Fig. S3A–C). However, *Tgm1* was not significantly different. In the IL-4-DMEM group, *Clcf1*, *Tgm1*, and *Emp1* were upregulated, whereas *Fbln5* and *Serping1* were inhibited, compared to the control group (Additional file 3: Fig. S3A–F). The above in vitro experiments suggested that culture from microglia promoted polarization of the A1 or A2 astrocyte subsets, which were used to conduct the following in vivo experiments of cell transplantation after SCI.

### Effect of A1 or A2 astrocyte on neuronal apoptosis and complexity

**Fig. 3:**
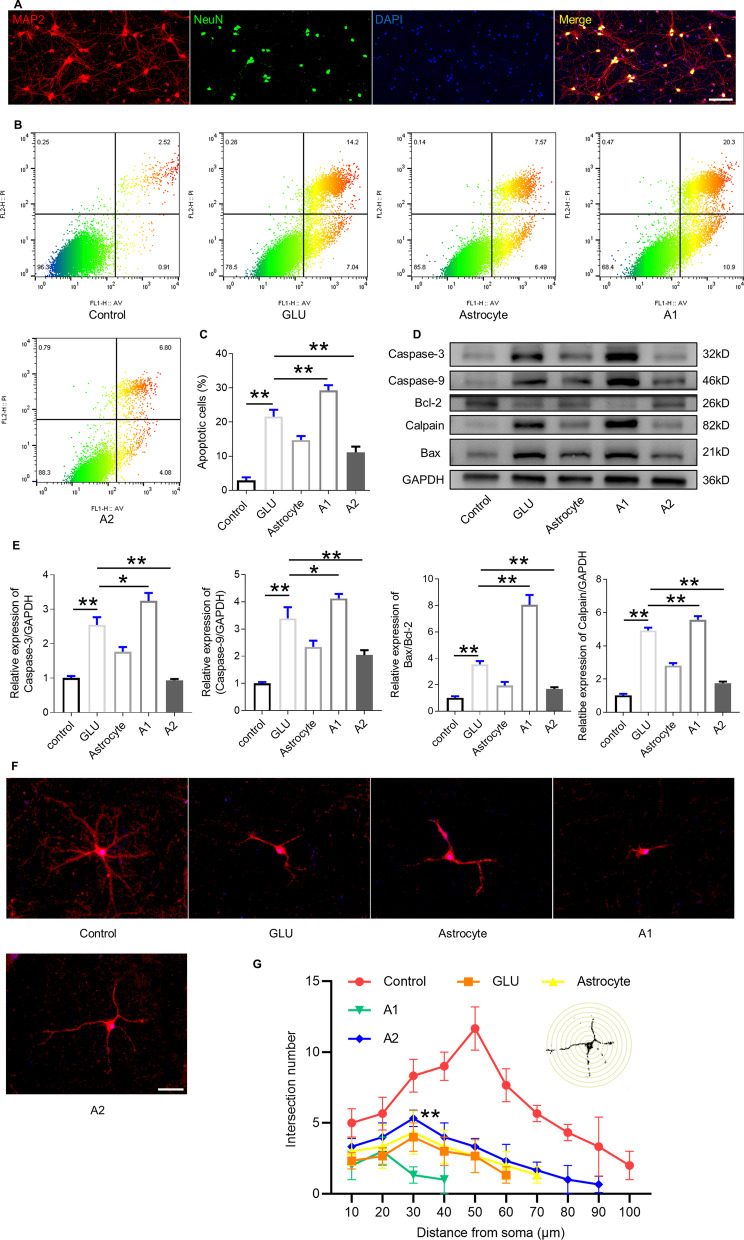
Effect of differentiated astrocyte on neuronal apoptosis and complexity. **A** Identification of neurons by MAP2 and NeuN immunofluorescence staining. Scale bar = 100 μm. **B** Representative images of flow cytometry assay results labeled with PI and annexin-V-FITC in primary neurons co-cultured with differentiated astrocytes after GLU-induced excitotoxicity. **C** Quantification of (**B**): percentage of apoptotic neurons in different groups. **D** Western blot analysis of apoptosis-related proteins in primary neurons. **E** Quantification of (**D**). **F** Representative images of MAP2 immunostaining for primary neurons in each experimental group. **G** Quantification of intersections by Sholl analysis. *p < 0.05, **p < 0.01

To further clarify the influence of differentiated astrocytes on neuronal apoptosis, which contributes to the neurological dysfunction induced by traumatic SCI, we co-cultured differentiated astrocytes and primary neurons (Fig. 3A). A high concentration of GLU was used to generate excitotoxicity to induce neuronal death and inhibit axonal sprouting, growth, and extension. Flow cytometry assay indicated a significantly increased number of apoptotic neurons after GLU treatment, which markedly decreased by culturing with A2 astrocytes. Moreover, primary astrocytes with no treatment inhibited the GLU-induced apoptotic effect, whereas A1 astrocytes further promoted neurotoxicity (Fig. 3B, C). Next, we analyzed apoptosis-related proteins such as proapoptotic proteins (Caspase 3, Caspase 9, Calpain and Bax) and antiapoptotic protein Bcl-2 by western blotting. Significantly elevated levels of Caspase 3, Caspase 9, Calpain and Bax were found in the primary neurons treated with GLU than in control. In contrast, Bcl-2 displayed a notably decreased level after GLU administration. After co-culturing A2 astrocytes with primary neurons treated with GLU, significantly lower expression of proapoptotic proteins and higher expression of Bcl-2 was detected than in the GLU group. In contrast, the GLU-treated neurons co-cultured with A1 astrocytes exhibited markedly increased levels of Caspase 3, Caspase 9, Calpain and Bax and reduced Bcl-2 level compared to the GLU group (Fig. 3D, E). Furthermore, as the key player in neuronal apoptosis, the Bcl-2/Bax ratio increased significantly in neurons co-cultured with A2 astrocytes, demonstrating A2 astrocytes’ neuroprotective function. To determine whether polarized astrocytes promoted the degree of neuron arborization, a Sholl analysis was conducted, which revealed an increased number of intersections from the radial distance to the cell soma, demonstrating a more complex dendritic arbor in the control group. However, in neurons treated with GLU, the number of intersections significantly decreased compared to that in neurons cultured with neurobasal medium alone. Additionally, neurons treated with A2 astrocytes showed a notable increase in the number of intersections when opposed to neurons in the GLU group, whereas A1 astrocytes exacerbated the impairment of synaptogenesis (Fig. 3F, G).

### Improvement of locomotor recovery with cell transplantation

**Fig. 4:**
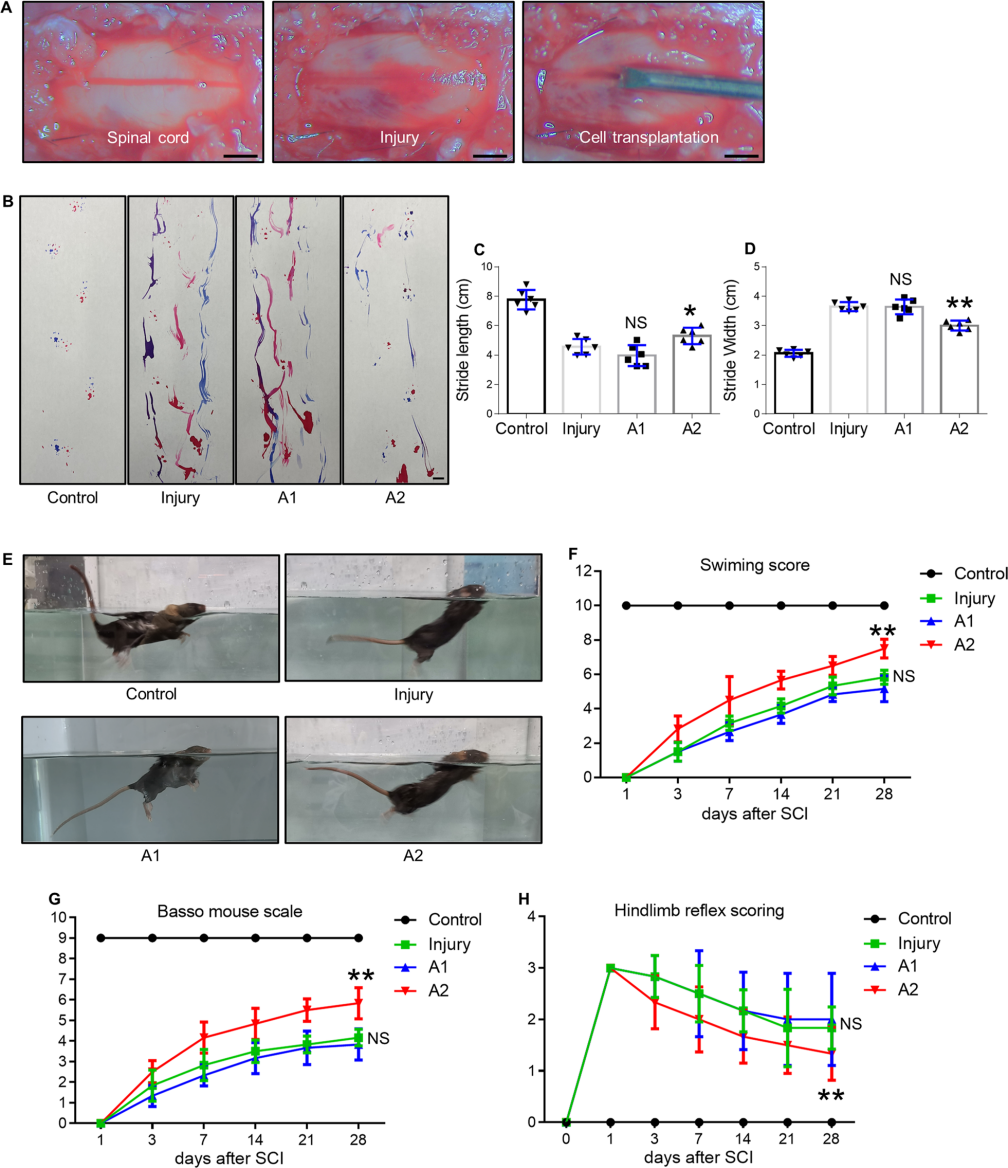
Assessment of locomotor recovery after cell transplantation. **A** Treatment schedule of astrocyte transplantation for the mouse model of spinal cord injury. **B** A footprint analysis performed at 28 dpi in control, spinal cord injury, A1 astrocyte-transplanted, and A2 astrocyte-transplanted groups. Scale bar = 1 cm. Quantification of the footprint analysis after SCI at 28 dpi by stride length (**C**) and width (**D**). **E** Representative images of the swimming tests in the four groups. **F** Quantification of (**E**) by the swimming score. **G** Time-dependent Basso mouse scale in SCI mice after hydrogel alone, A1 astrocytes wrapped in hydrogel, or A2 astrocytes wrapped in hydrogel at 1, 3, 7, 14, 21, and 28 dpi. **H** Time-dependent hindlimb reflex scoring in SCI mice after hydrogel alone, A1 astrocytes wrapped in hydrogel, or A2 astrocytes wrapped in hydrogel at 0, 1, 3, 7, 14, 21, and 28 dpi. Error bars show means ± SD (n = 6 in each group). *p < 0.05, **p < 0.01 compared to the injury group

A model of SCI was established in mice to illustrate the amelioration of motor defects with cell transplantation (Fig. 4A). To investigate whether differentiated astrocytes survived after implantation into mice after SCI, we transfected astrocytes using lentiviruses with green fluorescent protein (GFP) and identified the optimal multiplicity of infection (MOI) as 100 (Additional file 4: Fig. S4). Hydrogel was used to mix A1 astrocytes or A2 astrocytes and administrated at the SCI site. Hydrogel had little effect on cell survival of A1 astrocytes and A2 astrocytes (Additional file 5: Fig. S5). Although GFP-labeled astrocytes were detected on days 3, 7, and 14 post SCI, they were barely observed on day 28 (Additional file 6: Fig. S6A). The number of A1 and A2 astrocytes observed showed no significant difference (Additional file 6: Fig. S6B). A footprint analysis conducted at 28 dpi suggested that mice receiving an A1 astrocyte transplant had trouble taking consistent steps, which was no different from the untreated SCI mice (Fig. 4B). However, transplantation with A2 astrocytes significantly improved the walking function of mice, reflected by increased stride length and decreased stride width, compared to the A1 astrocyte-transplanted or untreated SCI mice (Fig. 4C, D). Swimming test experiments were conducted to further confirm the beneficial effect of A2 astrocytes on functional recovery. In accordance with the footprint analysis results, the swimming function was remarkably improved in the A2 group relative to the A1 astrocyte-transplanted or untreated SCI mice (Fig. 4E, F). Motor functional recovery of mice treated with A1 or A2 astrocytes was then evaluated using the widely accepted BMS and hindlimb reflex scoring (Fig. 4G, H). The score in the control group was in the normal range, whereas the other three groups displayed reduced scores after SCI. A remarkable variance in the scores between the three groups was found at 7 days, indicating that the transplantation of A2 astrocytes was effective in improving locomotor recovery (Fig. 4G). In addition, lower hindlimb reflex scoring in the A2 group clarified the positive influence of A2 on function restoration compared to that in the A1 group (Fig. 4H). In view of the importance of descending pathways for recovery after SCI, we next explored the spared supraspinal pathways by electrical stimulation in the motor cortex to elicit reproducible waveforms of EMG responses (Additional file 7: Fig. S7A). A2 astrocyte treatment dramatically increased the EMG response of the gastrocnemius muscle 6 weeks post SCI (Additional file 7: Fig. S7B, C).

### Assessment of lesion progression after cell transplantation

**Fig. 5:**
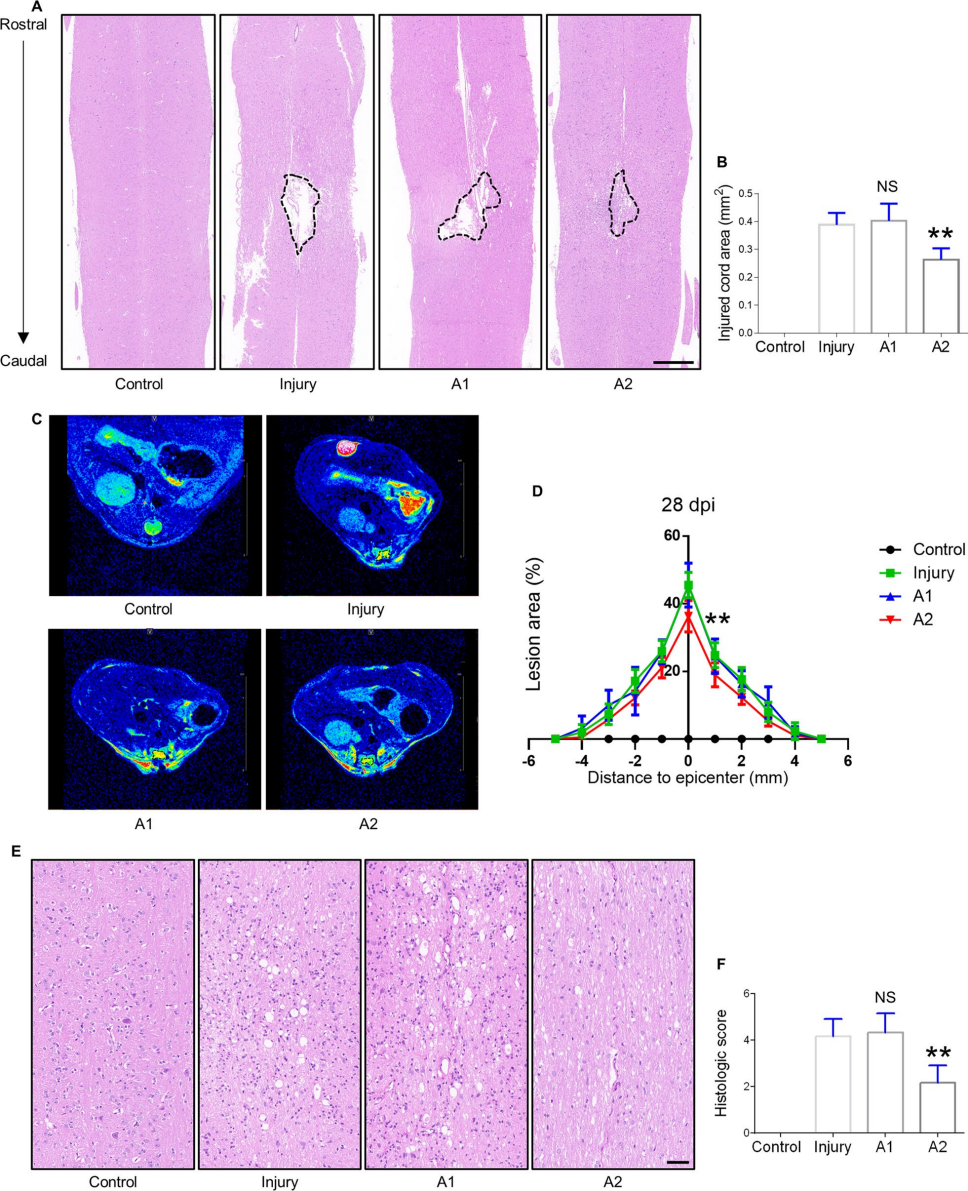
Histological evaluation of the injured site after spinal cord injury and astrocytes transplantation in mice. **A** H & E staining of the injured center of the spinal cord at 7 dpi. Scale bar = 500 μm. **B** Quantification of the area of the injured site of the spinal cord at 7 dpi. **C** Representative axial MRI images at 28 dpi in control, injury, A1 astrocyte-transplanted, and A2 astrocyte-transplanted groups. **D** Quantification of (**C**) by measuring the percentage of lesion area surrounding the center of lesion core. **E** H & E staining of the injured center of the spinal cord at 28 dpi. Scale bar = 50 μm. **F** Quantification of the histologic score of the injured site of the spinal cord at 7 dpi. Error bars show means ± SD (n = 6 in each group). **p < 0.01 compared to the injury group

Longitudinal sections of the mouse spinal cord at 7 dpi following cell transplantation were evaluated histologically by hematoxylin and eosin H & E staining. We found that A2 astrocyte transplantation significantly reduced the area of the injured cord. However, no significant difference was detected in the A1 astrocyte-transplanted group before and after transplantation (Fig. 5A, B). Morphological analysis of the injured cord area obtained on day 28 after SCI showed that A2 astrocyte transplantation significantly decreased the lesion area compared to untreated SCI mice, whereas A1 astrocyte had no therapeutic effect (Additional file 8: Fig. S8). Axial MRI images from the segment centered on the lesion epicenter were used to determine lesion progression. A direct comparison at 28 dpi displayed no remarkable differences in the lesion area between the A1 astrocyte-transplanted and untreated SCI mice. However, transplantation of A2 astrocytes significantly decreased the lesion area compared to transplantation of A1 astrocytes (Fig. 5C, D). The histologic score of H & E staining was quantified to assess tissue integrity, and we found that A2 astrocyte treatment showed lower histologic scores than A1 astrocyte treatment or no treatment after SCI (Fig. 5E–F).

### Effect of astrocyte transplantation on neural restoration and glia formation

**Fig. 6:**
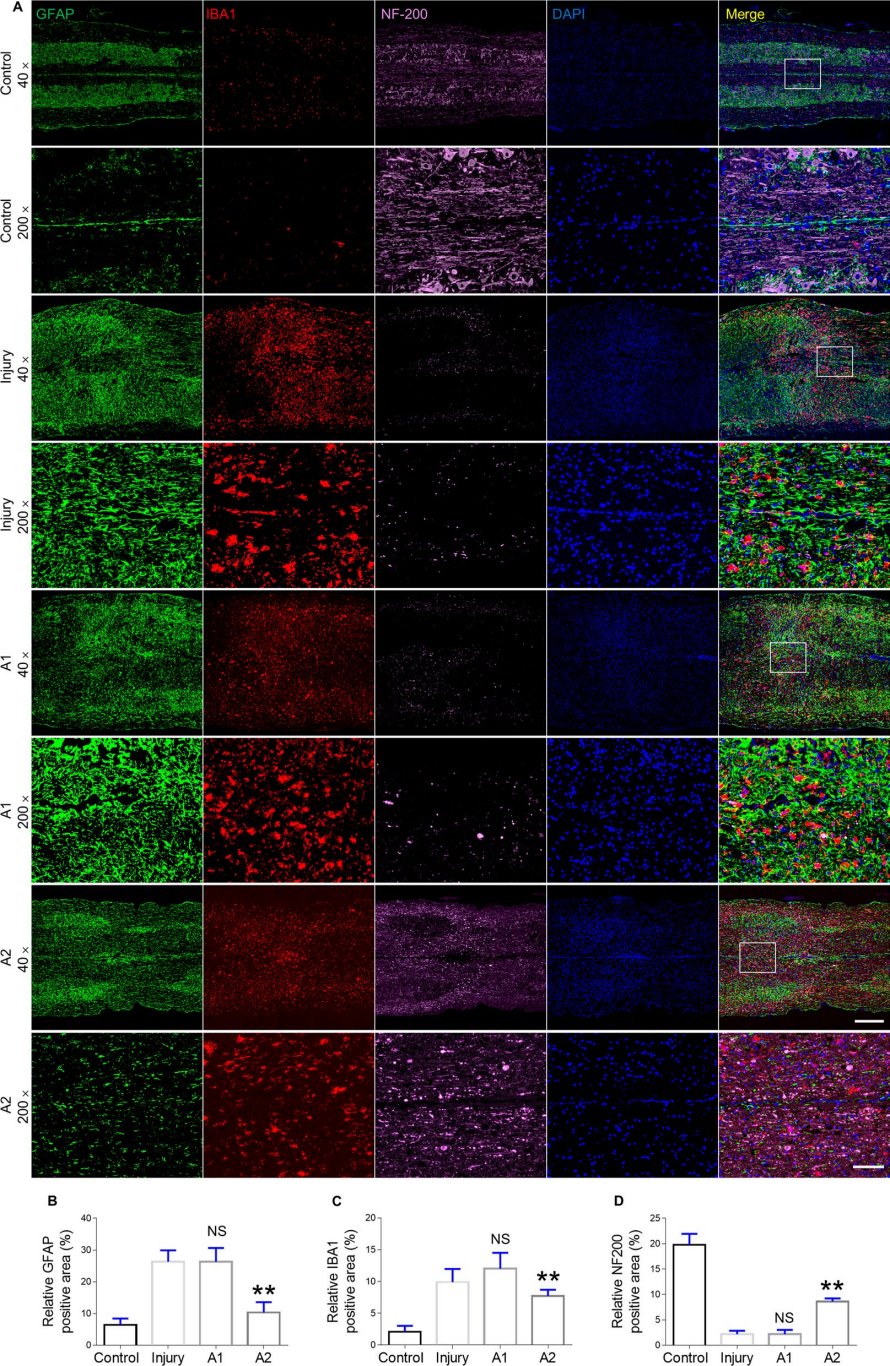
Glia formation and neural repair following astrocytes therapy in the SCI mouse model. **A** Immunofluorescence staining for GFAP (astrocyte marker: green), IBA-1 (microglia marker: red), NF-200 (neurofilament marker: pink), and DAPI (nuclei: blue) in the injury site of the spinal cord at 28 dpi after SCI and astrocyte transplantation treatment. Scale bar = 500 μm in 40 × images. Scale bar = 100 μm in 200 × images. **B** Quantification of the GFAP-positive area of the injured cord at 28 dpi. **C** Quantification of the IBA-1-positive area of the injured spinal cord at 28 dpi. **D** Quantification of the NF-200-positive area of the injured spinal cord at 28 dpi. Error bars show means ± SD (n = 6 in each group). **p < 0.01 compared to the injury group

Immunofluorescence staining for astrocytes (GFAP-positive), microglia (IBA-1 positive), and neurofilaments (NF-200-positive) were performed to assess tissue repair at 28 dpi. Microglia and astrocyte contribute to glial scars that impede neurofilament regeneration after SCI. Astrocyte proliferation was significantly elevated in the A1 astrocyte-transplanted mice than in A2 astrocyte-transplanted mice after SCI (Fig. 6A). Quantitative analysis of GFAP staining at the injury site also showed significant differences among the groups (Fig. 6B). Interestingly, transplantation of A2 astrocytes had an inhibitory effect on the accumulation of microglia (Fig. 6C). Quantitative analysis of IBA-1 positivity confirmed that the A2 astrocyte-transplanted mice displayed significantly less IBA-1 positivity than the A1 astrocyte-transplanted mice. Immunofluorescence staining for NF-200 identified an obvious population of neuronal cells in the control group (Fig. 6A). In other three groups of mice, although no integrated neurons were observed in the injury lesions, a significantly increased NF-200 positive area was noted in A2 astrocyte-transplanted mice than in the A1 astrocyte-transplanted and untreated SCI mice (Fig. 6D).

### Expression of A1 or A2 astrocyte markers in the injured spinal cord after transplantation

**Fig. 7:**
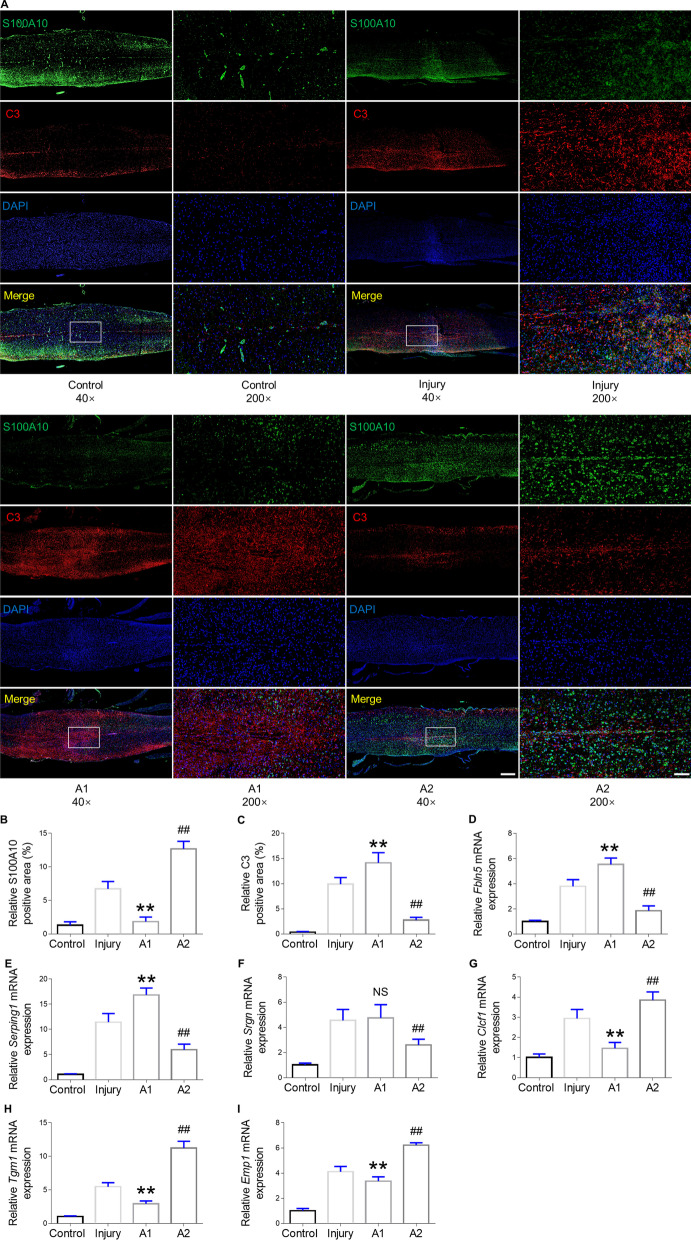
Identification of A1 and A2 astrocytes within the spinal cord 28 days post injury after cell transplantation. **A** Immunofluorescence staining for C3 (A1 astrocyte marker: red) and S100A10 (A2 astrocyte marker: green) in the injury site of the spinal cord at 28 dpi after SCI and astrocytes transplantation treatment. Scale bar = 500 μm at low magnification. Scale bar = 100 μm at high magnification. **B** Quantification of the S100A10-positive area of the injured spinal cord at 28 dpi. **C** Quantification of the C3-positive area of the injured spinal cord at 28 dpi. **D**–**F** RT-PCR analysis of A1-specific genes: *Fbln5*, *Serping1,* and *Srgn* relative expression and **G**–**I** A2-specific genes: *Clcf1*, *Tgm1*, and *Emp1* relative expression in the spinal cord at the injured level from control, injury, A1 and A2 groups. All mRNA expression was normalized to *GAPDH*. Error bars showed means ± SD (n = 6 in each group). **p < 0.01, ^##^p < 0.01 compared to injury group

Immunofluorescence staining for S100A10 and C3 was performed to determine whether transplantation influenced astrocytic differentiation in the spinal cord lesion area. Spinal cords with no injury showed low expression of S100A10 and undetectable C3. However, in the lesion area of the spinal cord at 28 dpi, expression levels of both C3 and S100A10 were increased, suggesting that A1 and A2 astrocytes were in homeostasis in response to injury (Fig. 7A). Moreover, expression of S100A10 was markedly enhanced in the A2 astrocyte-transplanted mice than in the A1 astrocyte-transplanted and untreated SCI mice (Fig. 7B). Conversely, the C3 level was enhanced in the A1 astrocyte-transplanted mice than in the A2 astrocyte-transplanted and untreated SCI mice (Fig. 7C). We next examined the specific gene expression in the astrocytes in control, injury, A1 and A2 groups. A1 astrocyte-specific genes, *Fbln5*, *Serping1,* and *Srgn,* were remarkably increased in the A1 group as opposed to the A2 group and injury group (Fig. 7D–F) whereas transplantation of A2 astrocytes markedly elevated the expression of *Clcf1*, *Tgm1*, and *Emp1* (A2 markers) (Fig. 7G–I).

### Evaluation of the demyelination of spinal axons following astrocyte transplantation after SCI

**Fig. 8:**
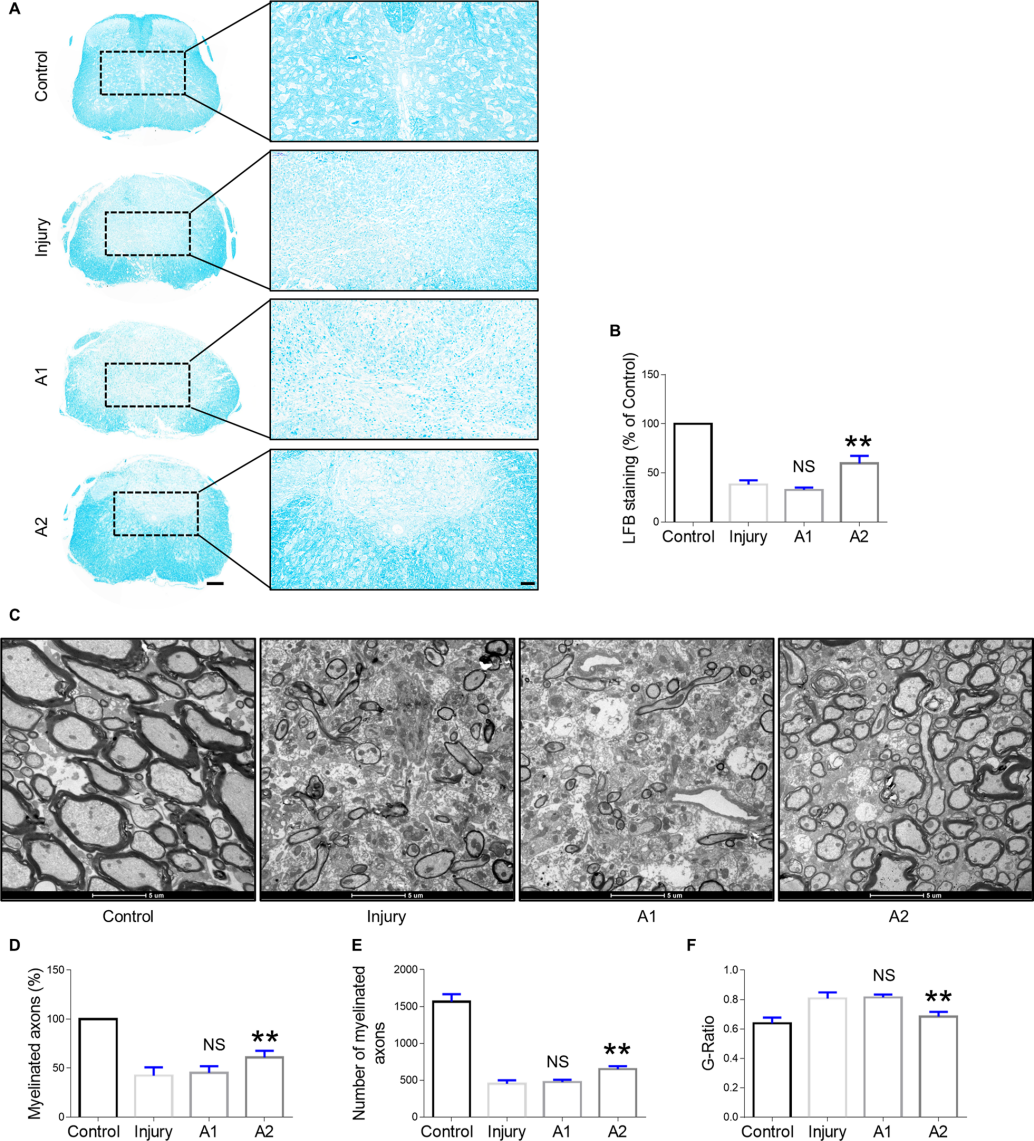
Demyelination analysis of spinal axons in SCI mice after treatment. **A** Luxol fast blue staining of axial sections of the epicenter from the lesion level at 28 dpi in control, SCI injury, A1 astrocyte-transplanted, and A2 astrocyte-transplanted groups. Scale bar = 200 μm at low magnification; scale bar = 50 μm at high magnification. **B** Quantification of Luxol fast blue staining in the injured area relative to the control group. **C** Electron micrographs of transverse sections of the spinal cord 28 days after spinal contusion injury in control, SCI injury, A1 astrocyte-transplanted, and A2 astrocyte-transplanted groups. Scale bar = 5 μm. **D** Percentage and **E** number of myelinated axons. **F** G-ratio in the four groups. Error bars show means ± SD (n = 6 in each group). **p < 0.01 compared to the injury group

To examine the effect of transplanting A1 or A2 astrocytes on myelination following SCI, tissue samples were stained with LFB and myelination was analyzed in the lesion site. Lesions of the mice treated with A2 astrocytes displayed significantly increased LFB staining compared to those of the mice transplanted with A1 astrocytes (Fig. 8A, B). These results were further validated by examining the myelin sheath of the injured site within the spinal cord via electron microscopy. The tissues in the uninjured group mice showed a highly organized structure of myelin sheath at 28 dpi, whereas those in the A1 astrocyte-transplanted and untreated SCI mice showed a lower number of myelinated axons and a higher extent of disorganized structures. However, in the A2 astrocyte-transplanted mice, there was a significantly higher percentage of myelinated and better-preserved axons (Fig. 8C–E). Moreover, a significant increase in the G-ratio was identified in the A1 astrocyte-transplanted and untreated SCI mice, indicating very thin myelin sheaths, whereas the A2 astrocyte-transplanted mice had a markedly decreased G-ratio (Fig. 8F), suggesting its beneficial effect on the preservation of myelination after SCI.

## Discussion

Astrocytes, as an abundant resident cell type in the CNS, play crucial roles in managing homeostasis by supplying neurotrophic support and sustaining synaptic integrity and function [31–35]. Astrocytes become reactive with a significant change in morphology, loss of normal astrocyte function, and alteration of gene expression under pathological circumstances [16, 36]. In the last decades, the biological and physiological importance of astrocytes has come into the limelight [37–40]. Thus, determining the mechanisms underlying astrocyte activity could unveil their potential therapeutic potential for treating neurological disorders. Previous studies have established that reactive astrocytes induced by neuroinflammation are termed A1 and A2, similar to M1 and M2 of microglia [16, 41]. After CNS injury, A1 astrocytes are neurotoxic and rapidly kill mature neurons and differentiated oligodendrocytes [16]. In Alzheimer's disease, A1 astrocytes were shown to be highly activated by amyloid-β in the degenerative site [42, 43]. In amyotrophic lateral sclerosis and the acute stage of multiple sclerosis, spinal motor neurons, and demyelinating plaques showed increased A1 astrocytes levels [44, 45]. In contrast, A2 astrocytes play a neuroprotective role in neural tissue repair by promoting synapse formation and facilitating axon growth [46, 47]. In this study, we found that LPS alone could not directly activate astrocytes in accordance with a previous finding [16], whereas the culture medium from microglia treated with LPS had the ability to differentiate astrocytes to A1 type with a high level of C3 protein. These results suggested that M1 microglia were essential for the induction of A1 astrocytes in vitro. Moreover, we polarized A2 astrocytes by culturing in the presence of the cellular supernatant of microglia treated with IL-4. In accordance with a previous finding, the co-culture model showed that A2 astrocytes demonstrated notable neuroprotective effects and promoted neuron sprouting.

In recent years, cell transplantation has been identified as one of the most promising therapies, aiming to replace dead cells and create a suitable environment for neural repair after SCI. Different cell types, including microglia, neural progenitors, and stem cells, have been transplanted; some of these studies are being applicated to phase I clinical trials [48]. Considering the carcinogenesis of some unexpected stem cells, our study design used astrocytes originating from the CNS to transplant into the site of SCI. To our knowledge, no data to date has been reported on the transplantation of astrocytes to treat SCI. On the basis of the protection of neuro from neurotoxin by restriction from astrocytes, A1 and A2 may be at homeostasis to exert a neuroprotective role at the early stage of SCI. A1 astrocytes are known to induce neurological damage, and A2 astrocytes show a neuroprotective effect. Therefore, in our study, selective A2 astrocyte transplantation was performed, which displayed a beneficial effect on the recovery of motor function than transplantation of A1 astrocytes.

Systemic intravenous injection of transplantation cells into the tail has its limitations. First, the transplanted cells need to pass through the blood–brain barrier to arrive at the lesion site of SCI, and second, injection may have side effects on other tissues and organs, resulting in a higher cell loss. Moreover, according to recent research, although direct transplantation causes more damage to the spinal cord than does intravenous administration, orthotopic injection is more efficient due to the requirement for a lower number of cells to be injected [20]. Thus, cellular orthotopic injection was performed to directly transport cells in situ. Astrocyte scars are fundamental to maintaining tissue integrity and forming limitation borders to restrict neurotoxins and defend undamaged CNS cells at the early stage following SCI. Subsequently, however, astrocyte-formed scars are widely recognized as the main cause for the failure of axon regeneration over the damaged area to establish neural connection between the level above and below the injury plane. Therefore, we conducted astrocyte transplantation immediately following SCI. The results obtained from the footprint analysis, swimming score, and BMS and hindlimb reflex scoring suggested that selective A2 astrocytes transplantation had a beneficial effect on motor function recovery after SCI. Furthermore, the results of electromyography uncovered more preserved spared supraspinal pathways after A2 astrocyte treatment. To further explore the influence of transplanting A2 astrocytes on neural tissue repair, we analyzed astrocyte scar formation and neurofilament regeneration. A smaller GFAP-positive area with more NF200 staining area was observed in the A2 astrocyte-transplanted group than in the A1 astrocyte-transplanted and untreated SCI groups, which further confirmed the neuroprotective role of A2 astrocyte transplantation. In the CNS, astrocytes are important for the physiological and pathological environment and interact with microglia to mutually regulate each other’s function [49, 50]. In the present study, we also found a decreased IBA-1-positive area after A2 astrocyte transplantation compared to A1 astrocyte transplantation and no therapy, which indicated that A2 astrocytes had the potential ability to inhibit reactive microglia accumulation. Moreover, increased A2 astrocyte marker S100A10 positive area was found in the A2 group with enhanced expression of A2-like genes such as *Clcf1*, *Tgm1*, and *Emp1*.

Remyelination is another key recovery process following CNS injury [51–53]. Myelin fails to regenerate in the progressive phase of SCI [54–56]. On the one hand, reactive astrocytes caused by CNS diseases limit the ability of oligodendrocyte progenitor cells to mature into myelinating oligodendrocytes [57, 58]. On the other hand, astrocytes also have beneficial effects in demyelinating diseases [59]. For instance, astrocytes promoted myelin regeneration in mice after deleting voltage-gated calcium channels [60]. In our study, elevated remyelination in the lesion site following SCI was observed after transplantation of A2 astrocytes, indicating the contribution of A2 astrocytes to the formation of mature myelinated oligodendrocytes.

Our study had some limitations. First, primary astrocytes with no immunogenicity were difficult to obtain. Second, the mechanisms underlying the potentially beneficial therapeutic effects of astrocytes are unknown. However, with the development of gene-editing technologies such as the CRISPR-Cas system [61–63], other types of cells may be able to be reprogrammed to A2 astrocytes.

## Conclusions

In summary, the findings of this study suggest A2 astrocytes can protect neurons and transplantation of A2 astrocytes increases neurofilament formation and improves motor function recovery of mice after SCI. Our data open avenues for the possible use of astrocyte transplantation in the treatment of SCI and identifies transplanting A2 astrocytes as an effective therapy.

## Supplementary Information

**Additional file 1: Fig. S1.** Analysis of microglial and astrocyte population after separation. **A** Flow cytometry assay results of cells in the lower layer. **B** Flow cytometry assay results of cells in the upper layer.

**Additional file 2: Fig. S2.** Immunofluorescent staining for GFAP and C3 after directly stimulated by LPS. Scale bars = 200 μm.

**Additional file 3: Fig. S3.** Gene Expression of Astrocytes after Treated with LPS-activated DMEM or IL-4-activated DMEM. **A**–**C** RT-PCR analysis of relative expression of A1-like genes: Fbln5, Serping1 and Srgn relative expression and **D**–**F** A2-like genes: Clcf1, Tgm1, and Emp1 relative expression in primary astrocytes after 3 days in the control, LPS-activated DMEM and IL-4-activated DMEM groups. Error bars showed means ± SD (n = 6 in each group). ^#^p < 0.05, **p < 0.01, ^##^p < 0.01 compared to control group.

**Additional file 4: Fig. S4.** Identification of optimal MOI of lentiviruses with GFP.

**Additional file 5: Fig. S5.** Cell survival of astrocytes in hydrogel detected by CCK-8 test reagent.

**Additional file 6: Fig. S6.** Analysis of GFP-positive astrocytes transplanted to the SCI mice. **A** Representative images of axial section of spinal cord on day 3, 7, 14, or 28 after SCI with A1 or A2 astrocytes transplantation. Scale bars = 100 μm. **B** Quantification of (**A**): number of GFP-positive cells. Error bars showed means ± SD.

**Additional file 7: Fig. S7.** Hindlimb EMG response at 6 weeks after SCI in different groups. **A** Schematic diagram indicates EMG potentials of contralateral gastrocnemius muscle was recorded after electrical stimulation in the motor cortex of mice at 6 weeks after SCI. **B** Representative examples of EMG potentials were recorded by motor-cortex stimulation in the control, injury, A1 astrocyte and A2 astrocyte treatment groups after SCI.

**Additional file 8: Fig. S8.** Morphological analysis of spinal cord on day 28 after SCI in different groups. **A** Representative images of spinal cord on day 28 post SCI with A1 or A2 astrocytes transplantation. **B** Quantification of (**A**): injured cord area. Error bars showed means ± SD. **p < 0.01, compared to A1 group.

## Data Availability

The datasets used and/or analyzed during the current study are available from the corresponding author on reasonable request.

## Abbreviations

SCI: Spinal cord injury
CNS: Central nervous system
LPS: Lipopolysaccharide
GLU: Glutamate
C3: Complement 3
GAPDH: Glyceraldehyde 3-phosphate dehydrogenase
GFAP: Glial fibrillary acidic protein
IBA-1: Ionized calcium-binding adapter molecule 1
MAP2: Microtubule-associated protein 2
NeuN: Neuronal nuclear protein
BMS: Basso mouse scale
EMG: Electromyography
H & E: Hematoxylin and eosin
MRI: Magnetic resonance imaging
LFB: Luxol fast blue
BMS: Basso mouse scale
GFP: Green fluorescent protein
MOI: Multiplicity of infection
BMS: Basso mouse scale
BMS: Basso mouse scale
