# Supplementary figures and images for: Transplantation of A2 type astrocytes promotes neural repair and remyelination after spinal cord injury
Source: Cell Commun Signal. 2023 Feb 16;21:37. doi: 10.1186/s12964-022-01036-6 (PMC9936716; doi:10.1186/s12964-022-01036-6)

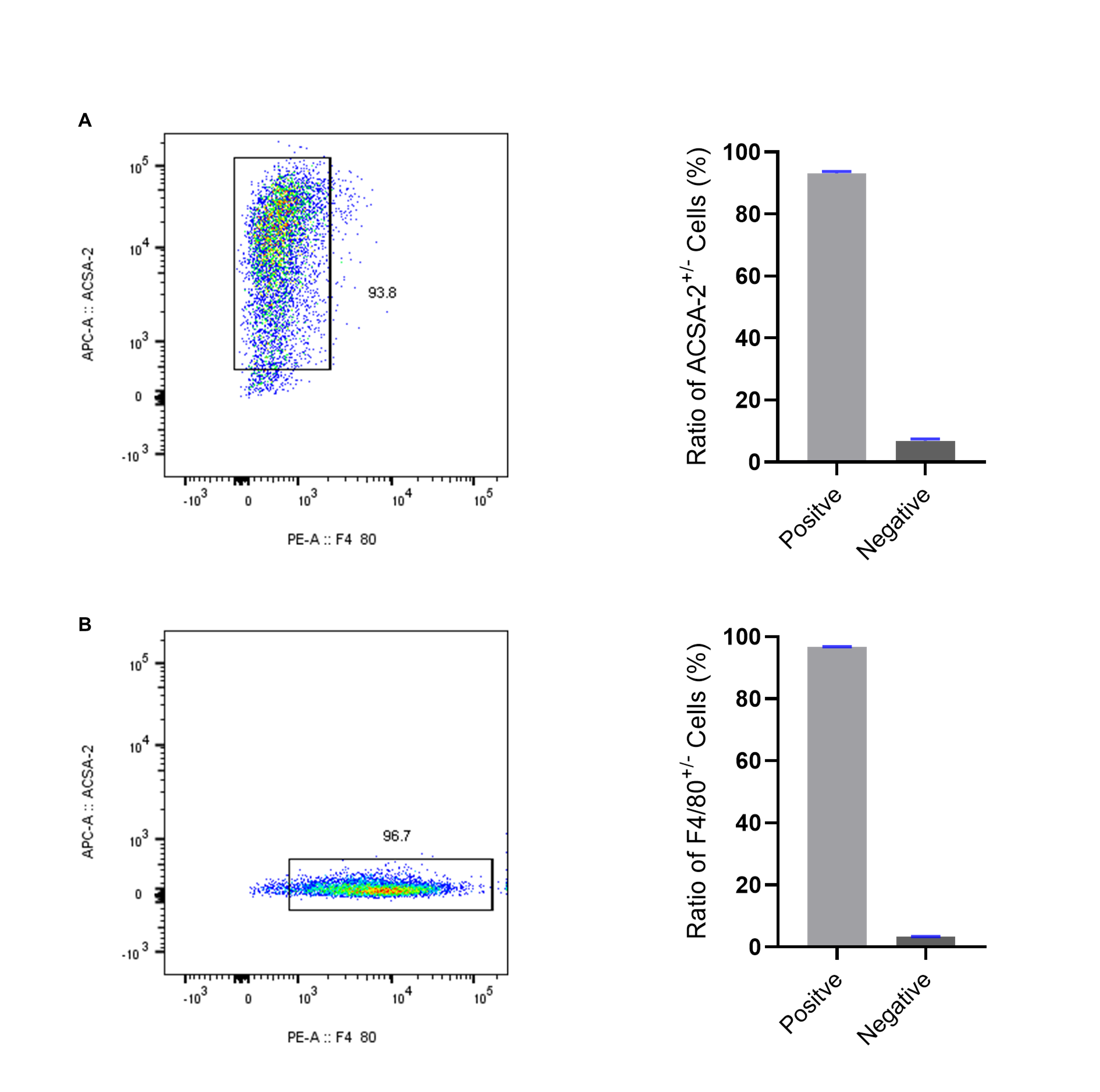

Supplement: Supplementary file 2 — Additional file 1: Fig. S1. Analysis of microglial and astrocyte population after separation. A Flow cytometry assay results of cells in the lower layer. B Flow cytometry assay results of cells in the upper layer. [file 12964_2022_1036_MOESM2_ESM.tif]

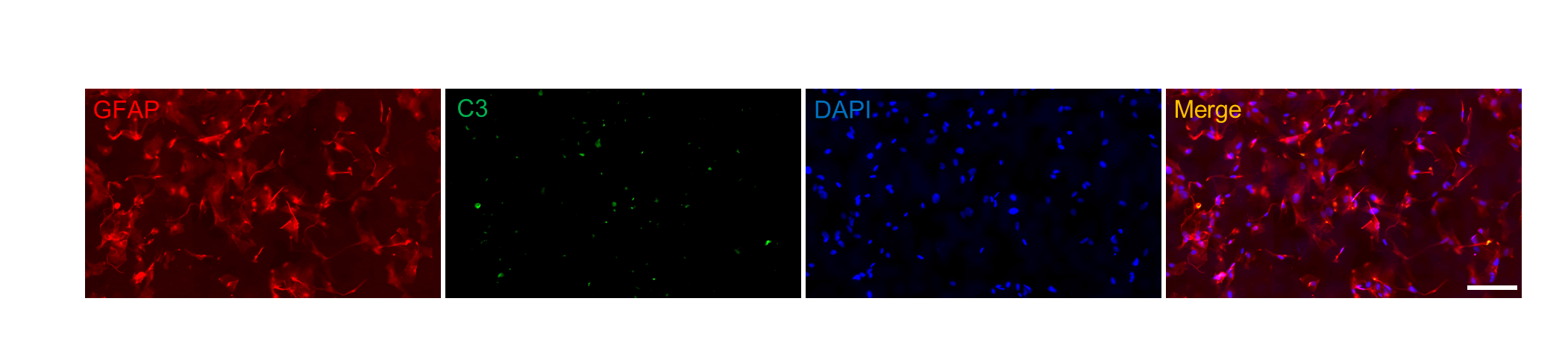

Supplement: Supplementary file 3 — Additional file 2: Fig. S2. Immunofluorescent staining for GFAP and C3 after directly stimulated by LPS. Scale bars = 200 μm. [file 12964_2022_1036_MOESM3_ESM.tif]

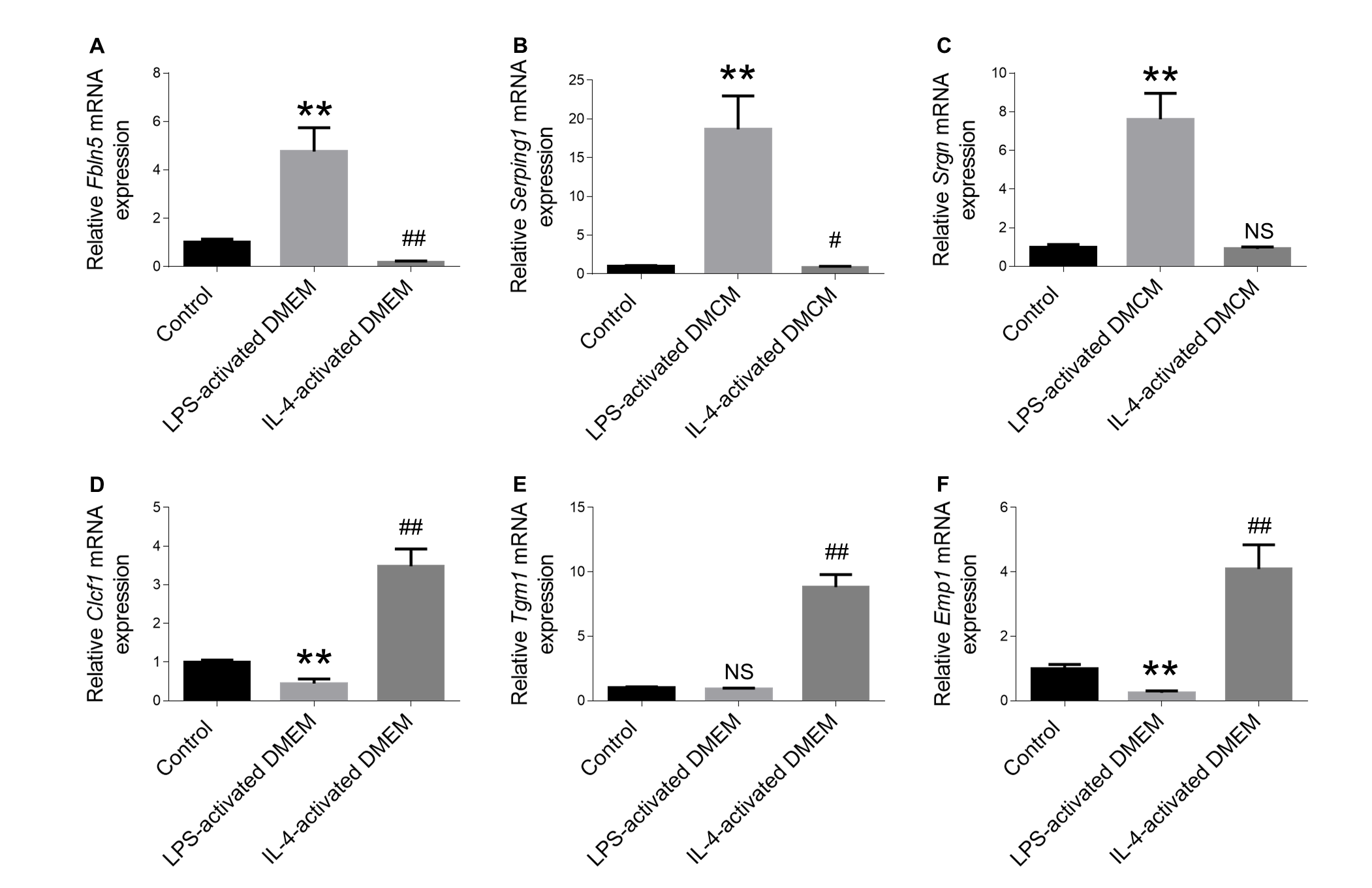

Supplement: Supplementary file 4 — Additional file 3: Fig. S3. Gene Expression of Astrocytes after Treated with LPS-activated DMEM or IL-4-activated DMEM. A–C RT-PCR analysis of relative expression of A1-like genes: Fbln5, Serping1 and Srgn relative expression and D–F A2-like genes: Clcf1, Tgm1, and Emp1 relative expression in primary astrocytes after 3 days in the control, LPS-activated DMEM and IL-4-activated DMEM groups. Error bars showed means ± SD (n = 6 in each group). #p < 0.05, **p < 0.01, ##p < 0.01 compared to control group. [file 12964_2022_1036_MOESM4_ESM.tif]

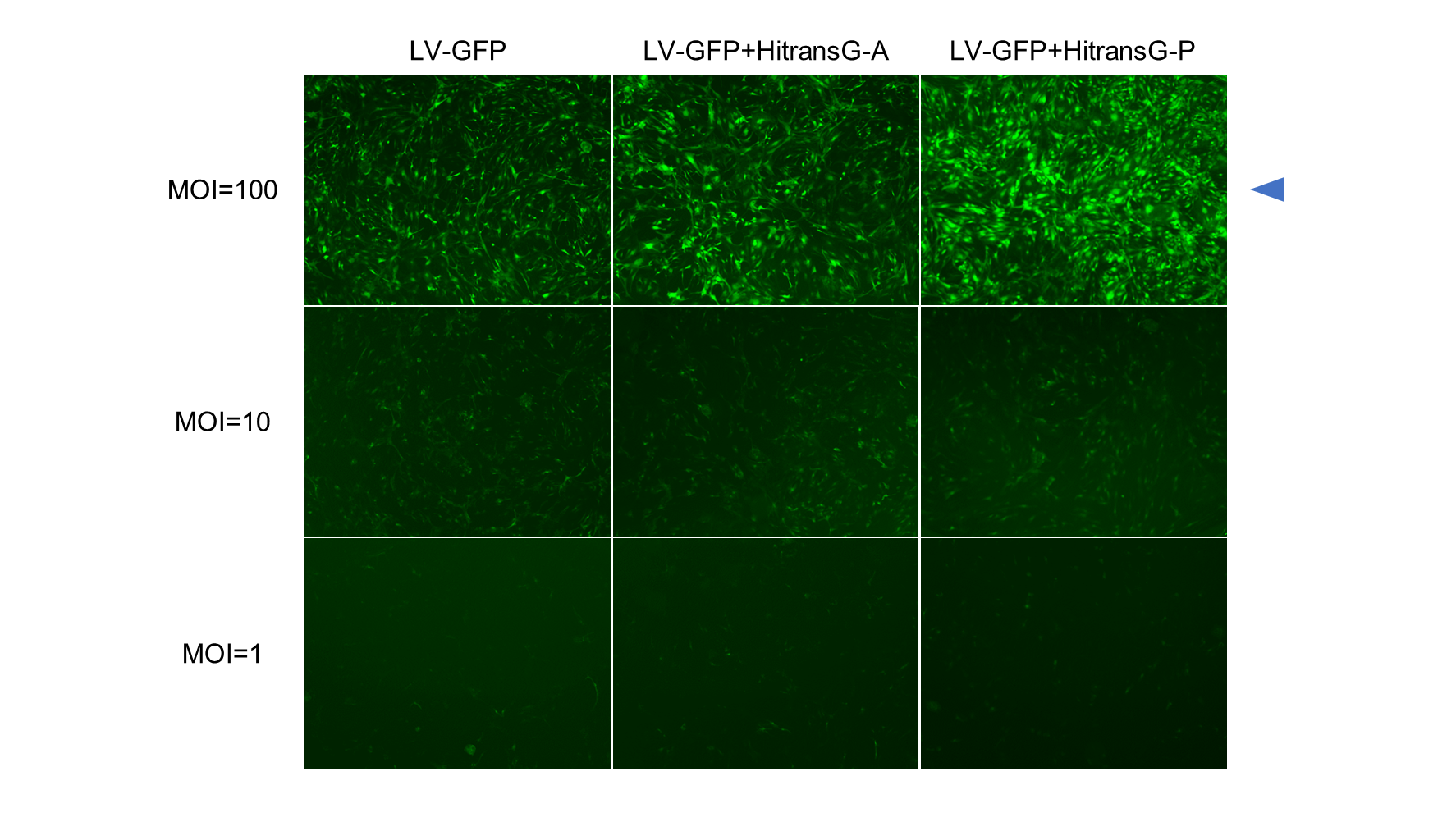

Supplement: Supplementary file 5 — Additional file 4: Fig. S4. Identification of optimal MOI of lentiviruses with GFP. [file 12964_2022_1036_MOESM5_ESM.tif]

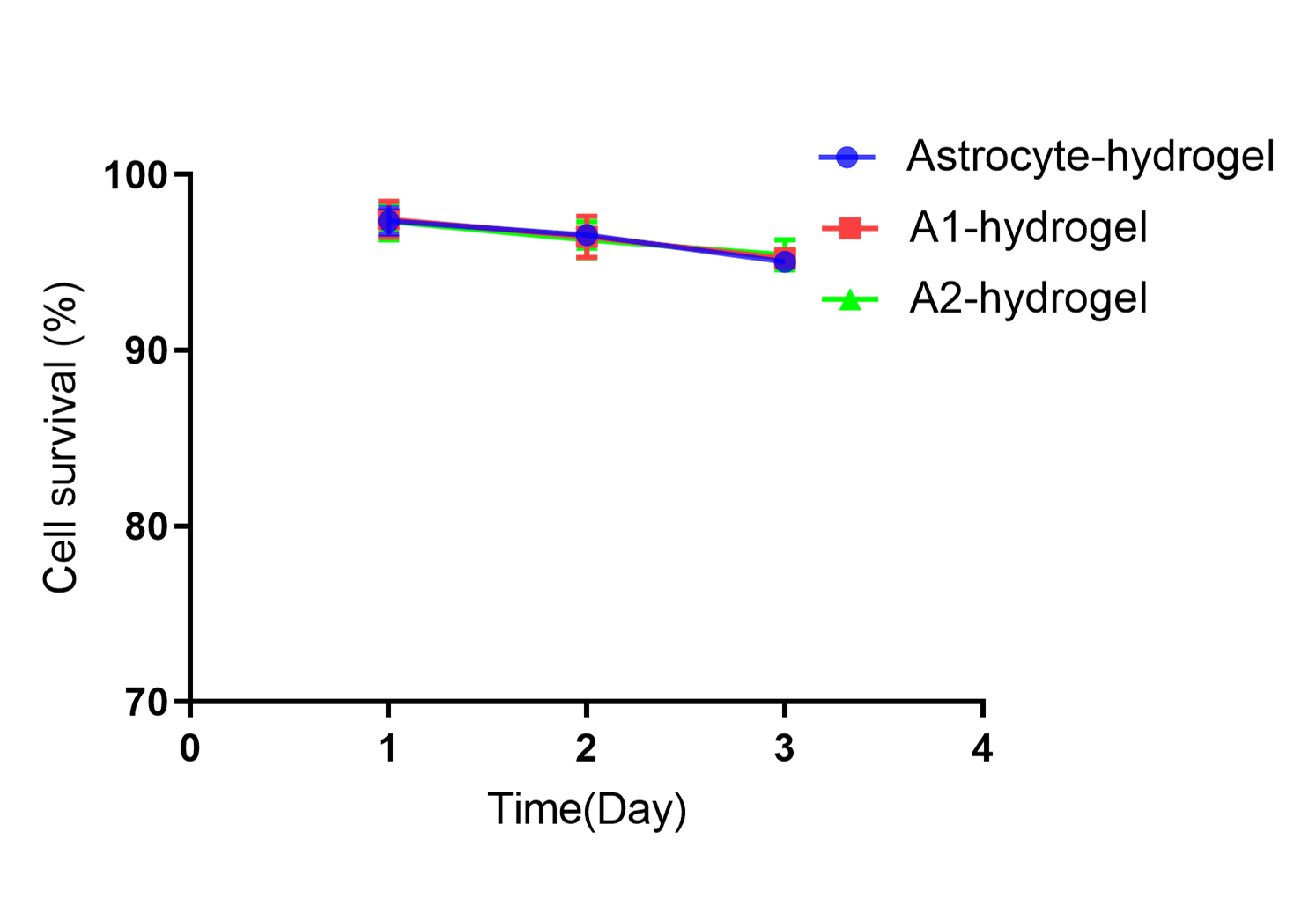

Supplement: Supplementary file 6 — Additional file 5: Fig. S5. Cell survival of astrocytes in hydrogel detected by CCK-8 test reagent. [file 12964_2022_1036_MOESM6_ESM.tif]

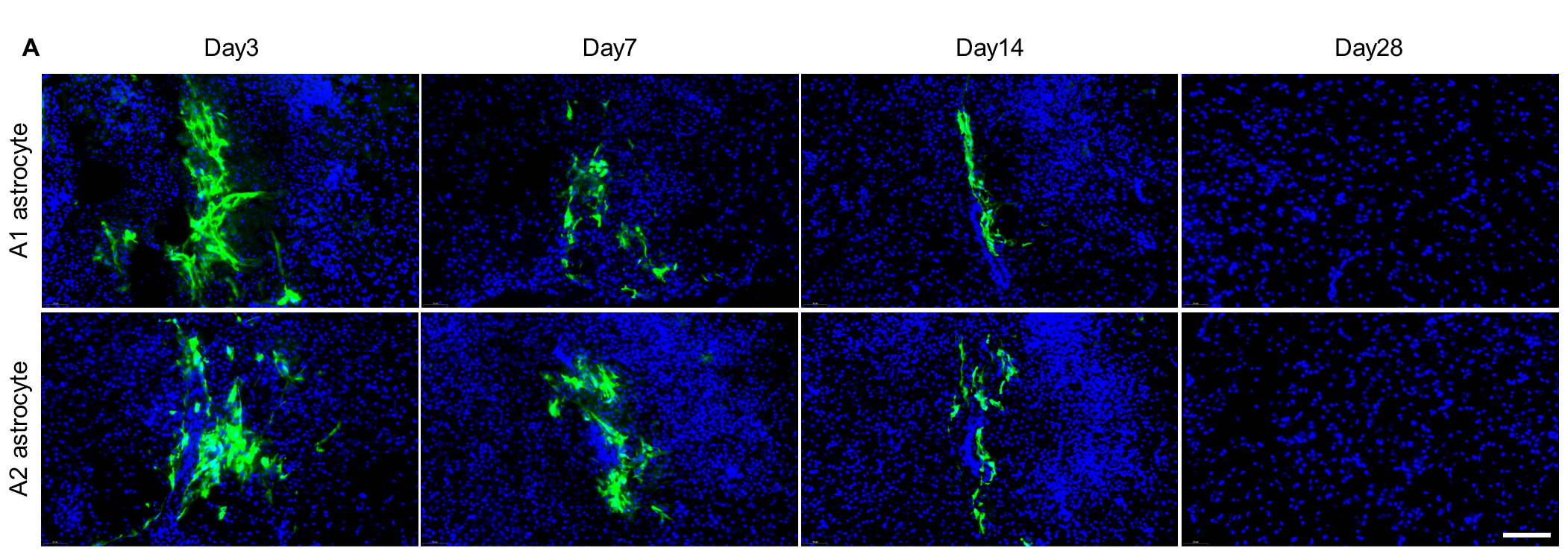

Supplement: Supplementary file 7 — Additional file 6: Fig. S6. Analysis of GFP-positive astrocytes transplanted to the SCI mice. A Representative images of axial section of spinal cord on day 3, 7, 14, or 28 after SCI with A1 or A2 astrocytes transplantation. Scale bars = 100 μm. B Quantification of (A): number of GFP-positive cells. Error bars showed means ± SD. [file 12964_2022_1036_MOESM7_ESM.tif]

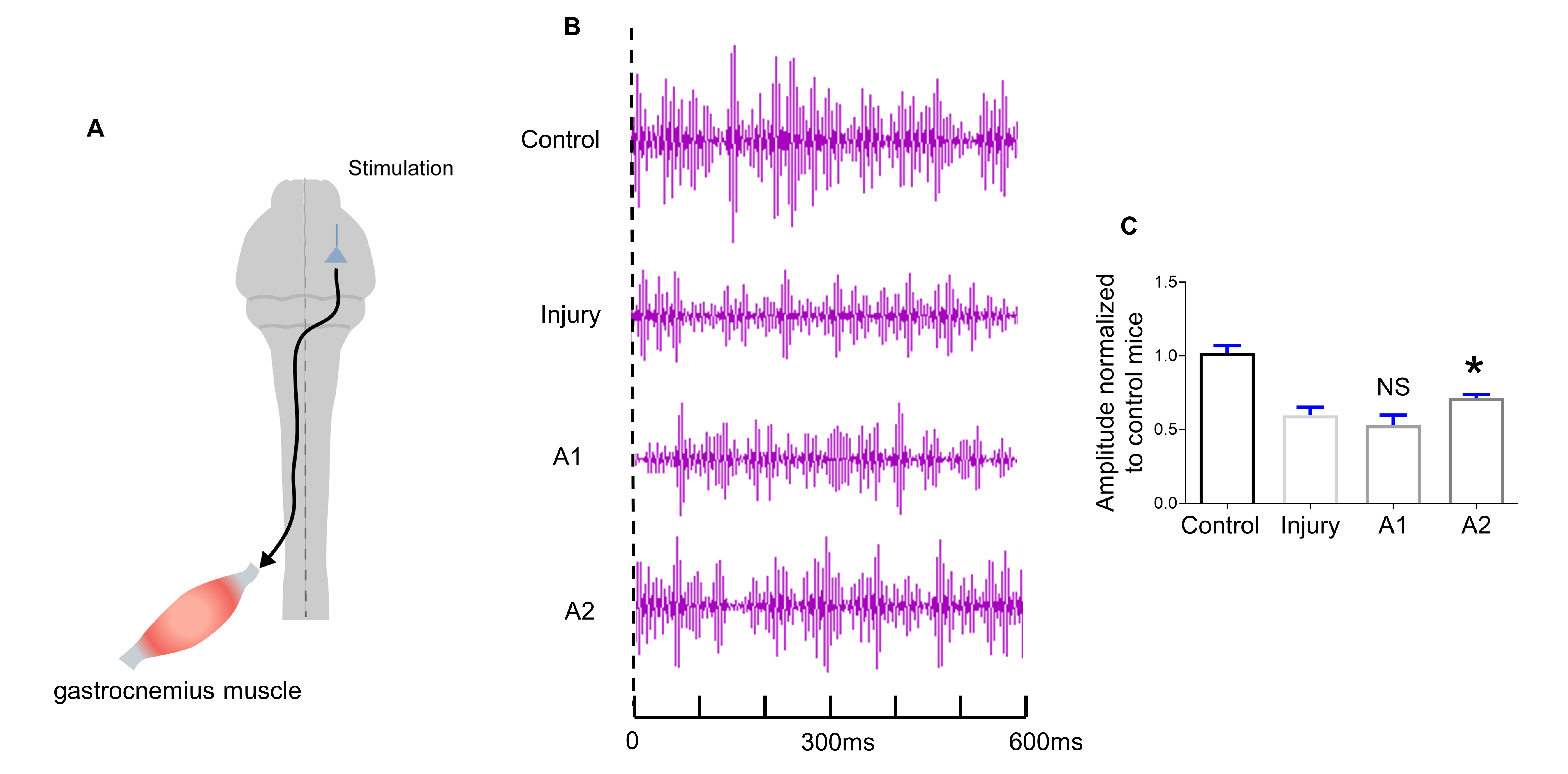

Supplement: Supplementary file 8 — Additional file 7: Fig. S7. Hindlimb EMG response at 6 weeks after SCI in different groups. A Schematic diagram indicates EMG potentials of contralateral gastrocnemius muscle was recorded after electrical stimulation in the motor cortex of mice at 6 weeks after SCI. B Representative examples of EMG potentials were recorded by motor-cortex stimulation in the control, injury, A1 astrocyte and A2 astrocyte treatment groups after SCI. [file 12964_2022_1036_MOESM8_ESM.tif]

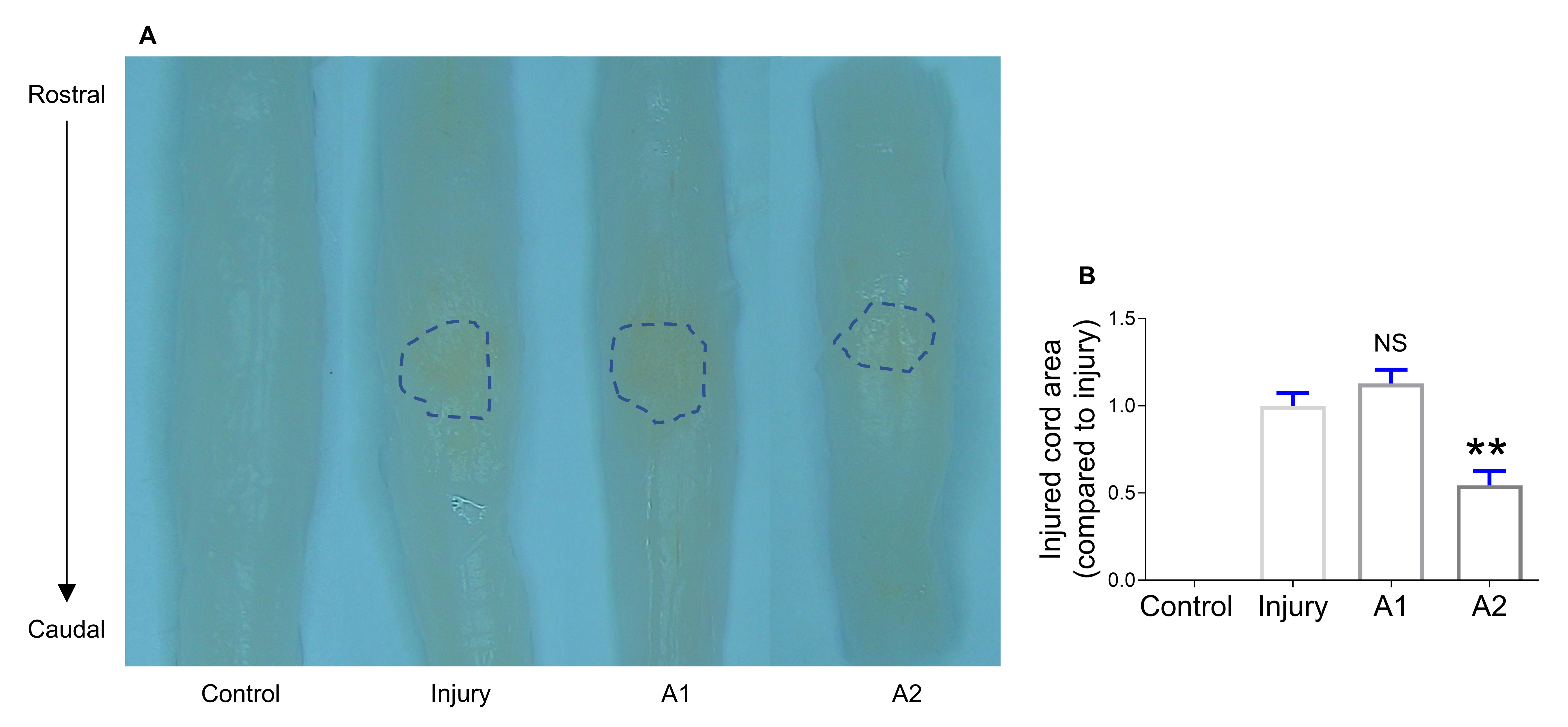

Supplement: Supplementary file 9 — Additional file 8: Fig. S8. Morphological analysis of spinal cord on day 28 after SCI in different groups. A Representative images of spinal cord on day 28 post SCI with A1 or A2 astrocytes transplantation. B Quantification of (A): injured cord area. Error bars showed means ± SD. **p < 0.01, compared to A1 group. [file 12964_2022_1036_MOESM9_ESM.tif]
